# The impact of signal-to-noise ratio, diffusion-weighted directions and image resolution in cardiac diffusion tensor imaging – insights from the ex-vivo rat heart

**DOI:** 10.1186/s12968-017-0395-x

**Published:** 2017-11-20

**Authors:** Darryl McClymont, Irvin Teh, Jürgen E. Schneider

**Affiliations:** 1 Division of Cardiovascular Medicine, Radcliffe Department of Medicine, University of Oxford, Oxford, UK; 2 Leeds Institute of Cardiovascular & Metabolic Medicine, University of Leeds, Leeds, UK

**Keywords:** Diffusion tensor imaging, Cardiac, Bias, Reproducibility, Helix angle, Sheetlet angle, Fractional anisotropy

## Abstract

**Background:**

Cardiac diffusion tensor imaging (DTI) is limited by scan time and signal-to-noise (SNR) restrictions. This invariably leads to a trade-off between the number of averages, diffusion-weighted directions (ND), and image resolution. Systematic evaluation of these parameters is therefore important for adoption of cardiac DTI in clinical routine where time is a key constraint.

**Methods:**

High quality reference DTI data were acquired in five ex-vivo rat hearts. We then retrospectively set 2 ≤ SNR ≤ 97, 7 ≤ ND ≤ 61, varied the voxel volume by up to 192-fold and investigated the impact on the accuracy and precision of commonly derived parameters.

**Results:**

For maximal scan efficiency, the accuracy and precision of the mean diffusivity is optimised when SNR is maximised at the expense of ND. With typical parameter settings used clinically, we estimate that fractional anisotropy may be overestimated by up to 13% with an uncertainty of ±30%, while the precision of the sheetlet angles may be as poor as ±31°. Although the helix angle has better precision of ±14°, the transmural range of helix angles may be under-estimated by up to 30° in apical and basal slices, due to partial volume and tapering myocardial geometry.

**Conclusions:**

These findings inform a baseline of understanding upon which further issues inherent to in-vivo cardiac DTI, such as motion, strain and perfusion, can be considered. Furthermore, the reported bias and reproducibility provides a context in which to assess cardiac DTI biomarkers.

**Electronic supplementary material:**

The online version of this article (10.1186/s12968-017-0395-x) contains supplementary material, which is available to authorized users.

## Background

Diffusion tensor imaging (DTI) allows for probing tissue microstructure. In the heart, it is increasingly being used as a non-invasive method of characterising healthy, as well as diseased hearts, such as those with hypertrophy or myocardial infarction [1, 2]. DTI minimally requires six diffusion-weighted (DW) images, in addition to one non-DW image, and fitting a diffusion tensor to each voxel [3]. In cardiac DTI, the primary, secondary and tertiary eigenvectors of this tensor, **v**_1_, **v**_2_ and **v**_3_, are generally thought to correspond to the locally prevailing cell long-axis, sheetlet, and sheetlet-normal directions respectively [4]. The primary eigenvalue is typically considerably larger than the other two eigenvalues, making the estimation of **v**_1_ relatively robust. However, reliable sorting of **v**_2_ and **v**_3_ in the presence of noise, and therefore reliable estimation of sheetlet angles, remains a challenge [5].

Invariably, trade-offs are made particularly in clinical DTI scans between the spatial resolution, the number of DW directions (ND), and the number of signal averages (NSA) to improve the signal-to-noise ratio (SNR). It is well recognised that increasing ND yields less biased estimates of DTI parameters, albeit with diminishing benefits for ND larger than 30 [6]. However, in most cardiac in-vivo studies, ND ranges between 6 and 12 [7–13]. Ignoring non-DW scans, the total scan time is directly proportional to ND and NSA, while SNR is proportional to the square root of NSA.

The effects of noise in diffusion cardiovascular magnetic resonance (CMR) are well established, with Rician noise raising the noise floor and causing underestimation of diffusion coefficients and biasing anisotropy measures [14]. These effects can be mitigated by phasing the data to produce normally distributed noise [10, 15], though such methods require accurate phase maps and are not yet widely utilised. In cardiac in-vivo studies, SNR is typically between 26 and 34 in the non-DW images after averaging [9, 10, 12]. The SNR in DW images will invariably be lower than non-DW images as a result of the diffusion attenuation. Higher diffusion weighting yields greater contrast with respect to the non-DW image but lowers SNR, and as such DTI is subject to an additional trade-off between accuracy and precision with respect to the choice of b-value [10].

Imaging resolution, or resolution as is referred to herein, is also subject to a trade-off with acquisition time, as well as with SNR. In general, increasing imaging resolution will decrease SNR, as the majority of the energy of the signal is concentrated in the low frequency components, whereas the energy of white noise is distributed across all frequencies. Human clinical DTI is typically acquired with an in-plane resolution of 2.7 mm [7–12], and interpolation is common [9–11]. Slice thickness ranges from 6 to 10 mm [8, 11, 13], with 8 mm being most commonly employed [7, 9, 10, 12].

In this work we modify high quality DTI datasets of ex-vivo rat hearts in terms of ND, SNR, and resolution, to establish their effect on the accuracy and precision of DTI parameters. Ex-vivo data is used in order to separate these effects from those of other confounding factors, such as motion, strain, perfusion, and partial voluming inherent in lower resolution clinical data [16]. The results are however equally relevant to both the ex-vivo and clinical setting.

## Methods

Experimental investigations conformed to the UK Home Office guidance on the Operations of Animals (Scientific Procedures) Act 1986 and were approved by the University of Oxford ethical review board. Five hearts were excised from Sprague-Dawley rats during terminal anaesthesia. Isolated hearts were swiftly perfused in Langendorff constant pressure mode with modified Krebs-Henseleit solution, cardioplegically arrested in a relaxed diastolic-like state with high potassium and perfused with low osmolality Karnovsky’s fixative doped with 2 mM gadolinium (Gd) complex Prohance (Bracco, Minnesota, USA). The hearts were then immersed in 50 mL of the same fixative and kept at 4 °C to ensure complete distribution of fixative and Gd. The median time and interquartile range from fixation to scanning was 23 and 7.5 days respectively. Prior to imaging, samples were rinsed three times in PBS + 2 mM Gd, and embedded in 1% agarose gel (Web Scientific, Crewe, UK) in PBS + 2 mM Gd to avoid sample motion and gradients in osmolality and contrast agent concentration.

Non-selective 3D fast spin echo DTI data were acquired on a 9.4 T preclinical MRI scanner (Agilent, California, USA) with a shielded gradient system (max gradient strength = 1 T/m, rise time = 130 μs), and transmit/receive birdcage coil (inner diameter = 20 mm; Rapid Biomedical, Rimpar, Germany). Acquisition parameters were: repetition time (TR) = 250 ms, echo time (TE) = 9.3 ms, echo spacing = 4.9 ms, echo train length (ETL) = 8, field-of-view (FOV) = 20 × 16 × 16 mm, resolution = 100 × 100 × 100 μm, number of non-DW images = 8, number of DW directions = 61, b_effective_ = 1000 s/mm^2^, diffusion duration (δ) = 2 ms, diffusion time (Δ) = 5.5 ms, receiver bandwidth = 100 kHz. The total acquisition time was 15.3 h. Temperature was assessed in one heart with a calibrated thermistor. The peak temperature measured was 23.8 °C, and temperature fluctuations were within 1 °C. [17].

The DW and non-DW images were acquired with optimised receiver gain settings [17], resulting in the images having different noise intensities. Separate noise-only datasets were acquired for both gain settings, using the same sequence without radiofrequency pulses [18] and with a shorter TR of 67 ms. The noise level, *σ*_acquisition_, was measured as the standard deviation of the real channel of the noise data in the image domain. The SNR of the data is defined as SNR = S/*σ*_acquisition_, where S is the mean myocardial signal intensity. The eight non-DW images were combined using complex averaging prior to processing.

**Fig. 1:**
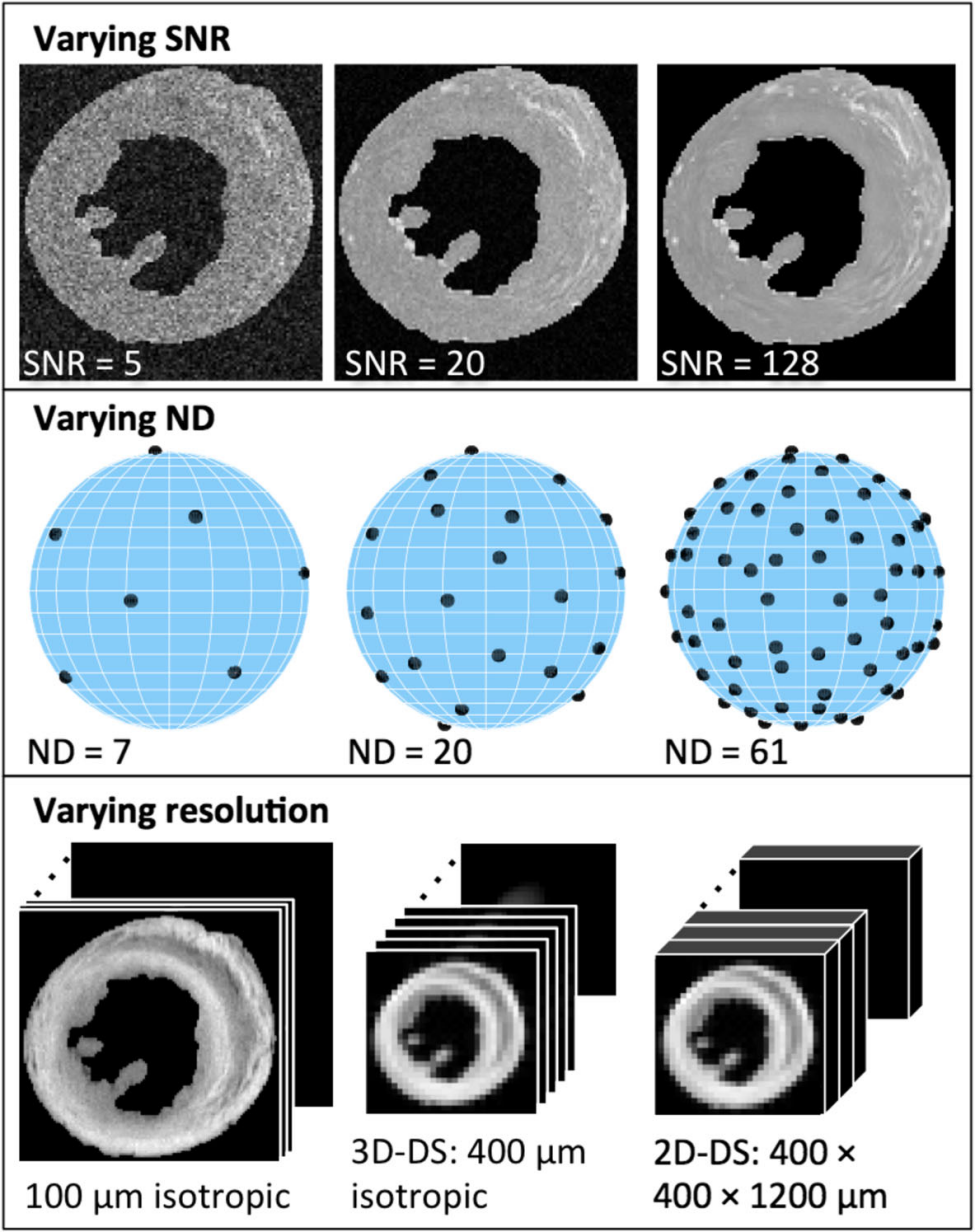
Illustration of the proposed methodology. Top: The SNR was varied by adding complex white noise to the data. Magnitude images were passed to the tensor fitting algorithm. Middle: The diffusion encoding scheme allowed for the truncation of directions while maintaining an approximately uniform distribution. A dataset with the desired number of directions can be generated by selecting the first ND images. Bottom: The resolution was varied by either truncating the 3D k-space data (3D–DS), or by first applying slice-selection and then truncating the 2D k-space data (2D–DS)

Examples of varying the resolution, number of DW directions and SNR are presented in Fig. 1. The effective image resolution was increased by truncating the data in k-space. As a human heart is approximately 6–8 times larger in each dimension than a rat heart, a resolution of 400 μm was chosen to approximately match the currently reported clinical human in-plane resolution of 2.7 mm. Two down-sampling schemes were considered: one mimicking a 3D acquisition with isotropic 400 μm resolution (3D–DS), and one mimicking a 2D multi-slice acquisition, with anisotropic voxels with 400 μm resolution in-plane and a slice thickness of 1.2 mm (2D–DS). In the 2D–DS scheme, the resolution was decreased in the slice-selection direction by averaging the complex data over the slice thickness. The slice thickness was chosen to proportionally match the 6–10 mm slice thickness in human DTI. This resolution is slightly larger than the 350 μm resolution in a recent in-vivo study of DTI in rats by Welsh et al. [19], but the slice thickness is lower than the 3 mm employed in that study. The net result is that the volume of each voxel (0.19 mm^3^) in the 2D–DS scheme is approximately half the volume of the voxels in the study of Welsh et al. (0.36 mm^3^). The SNR of the down-sampled data was higher than in the acquired data by a factor of 8 (i.e. (4 × 4 × 4)^0.5^) in the 3D–DS scheme, and 13.9 (i.e. (4 × 4 × 4 × 3)^0.5^) in the 2D–DS scheme [20].

The sixty-one DW directions were specified using an encoding scheme [21] wherein the number of directions, ND, may be reduced while maintaining an approximately uniform spherical distribution for ND ≥ 7. The truncated data is simply defined as the (averaged) non-DW image and the first ND DW images, for ND between 7 and 61.

The SNR was varied by adding normally distributed noise independently to the real and imaginary channels of the images. The mean of the noise was zero, and the variance ${\sigma}_{\mathrm{added}}^2$ was computed according to the relationship: ${\sigma}_{\mathrm{desired}}^2={\sigma}_{\mathrm{acquisition}}^2+{\sigma}_{\mathrm{added}}^2$. Note, the simulated SNR refers to that of the non-DW images (SNR_non-DW_) in all experiments, and the variance of the noise has been kept constant between the non-DW and DW images.

Ground truth diffusion tensors were derived from all 61 DW-images with no added noise. Magnitude data was passed to the tensor computation routine, which solved the DT equation S_DT_ = *S*_0_ exp(−BD) using linear least squares regression. The principal eigenvalues *λ*_1_, *λ*_2_, and *λ*_3_, mean diffusivity (MD), fractional anisotropy (FA), helix angle (HA), transverse angle (TA), sheetlet elevation (SE), sheetlet azimuth (SA), and secondary eigenvector angle (E2A) were derived from the tensor. In order to facilitate comparison to recent studies reporting the E2A, only the magnitude of this angle is reported, ignoring angle polarity. The parametric angles were defined relative to a local coordinate system, as described by Teh et al. [17]. The left ventricle was segmented using an in-house semi-automatic segmentation tool, excluding the papillary muscles from the segmented volume.

### Experiment 1: Effects of image resolution

Experiment 1 involved comparing the ground truth DW parameters to the images with lower resolution. The 17-segment American Heart Association model [22] was used for HA quantification. A transmural profile in a single slice in the centre of segments 1–16 was plotted, and the range and linearity (R^2^) was calculated. The angles were unwrapped prior to analysis. Segment 17 was excluded from this quantification, as the helix angle is not expected to follow the standard left-handed to right-handed profile at the apex. In order to preserve the location of the transmural profiles, the down-sampled images were interpolated to match the ground truth images. The interpolation kernel for the 3D–DS images was a sinc function (i.e. equivalent to zero-padding in k-space without low-pass filtering) in all three dimensions, whereas the 2D–DS images utilized linear interpolation in the slice selection direction and sinc interpolation in the short-axis plane.

### Experiment 2: Effects of ND and SNR

Experiment 2 fitted a diffusion tensor to images with variable ND and SNR_non-DW_ in order to elucidate the relationship between these factors and the accuracy and precision of DTI parameters. The 2D–DS scheme was used, as clinical diffusion imaging is currently almost exclusively performed using 2D imaging. The ground truth images were also resampled to the 2D–DS resolution as described above, but had no added noise and ND = 61. The accuracy of the parameters was computed from the mean of the difference between the parameters derived from the 2D–DS data, and the ground truth. These values were averaged over all myocardial voxels, and across the five hearts. Similarly, precision was given by the standard deviation of the difference between the estimated parameters and the ground truth. Circular statistics were used in the case of the parametric angles. The theoretical scan time for each combination of ND and SNR was assumed to be proportional to ND × SNR^2^ (i.e. assuming that increased SNR is achieved exclusively through repeated signal averaging).

Data and models will be made available upon request.

## Results

The median (and interquartile range) SNR of the acquired non-DW over five hearts was 39.5 (0.2), which increased to 111 (0.6) following complex averaging of the non-DW images. The median SNR of the DW images was 24.5 (0.2). The median values of the DTI parameters in the ground truth left ventricle are as follows: $$ {\displaystyle \begin{array}{l}{\lambda}_1=1.37\kern0.5em (0.08)\times {10}^{-3}{\mathrm{mm}}^2/\mathrm{s},\kern0.5em {\lambda}_2=0.98\kern0.5em (0.02)\times {10}^{-3}{\mathrm{mm}}^2/\mathrm{s},\kern0.5em \\ {}{\lambda}_3=0.86\kern0.5em (0.02)\times {10}^{-3}{\mathrm{mm}}^2/\mathrm{s},\kern0.5em \mathrm{MD}=1.07\kern0.5em (0.02)\times {10}^{-3}{\mathrm{mm}}^2/\mathrm{s},\kern0.5em \mathrm{FA}=0.25\kern0.5em (0.01),\kern0.5em \left(\mathrm{n}=5\right)\end{array}} $$.

### Experiment 1: Effects of image resolution

Using the 3D–DS scheme, the median *λ*_1_ was 0.87% lower, *λ*_2_ was 0.87% lower, and *λ*_3_ was 0.16% lower than in the high resolution data, resulting in 0.69% lower MD and 2.35% lower FA. Using the 2D–DS scheme, the median *λ*_1_ was 2.20% lower, *λ*_2_ was 0.21% lower, and *λ*_3_ was 0.77% higher than in the high resolution data, resulting in 0.77% lower MD and 7.41% lower FA.

Table 1 presents the range and linearity of representative HA profiles in the first 16 segments of the American Heart Association 17-segment model. In general, the linearity of the high-resolution data was very high, with the majority of segments having a median linearity of above 0.95. The lower resolution protocols lead to slightly more linear helix angle profiles, with a mean increase in linearity of 0.015 and 0.016 in the 3D–DS and 2D–DS schemes, respectively.

**Table 1:** Range and linearity of transmural profiles of the helix angle

Region	100 μm 3D		400 μm 3D–DS		400 μm 2D–DS	
	Range (°)	Linearity	Range (°)	Linearity	Range (°)	Linearity
1	102	0.92	94	0.93	87	0.93
2	170	0.99	167	0.99	166	0.99
3	133	0.98	136	0.97	130	0.97
4	116	0.90	104	0.96	92	0.97
5	114	0.98	109	0.99	108	0.99
6	143	0.88	117	0.97	113	0.98
7	114	0.98	115	0.98	114	0.98
8	154	0.99	144	0.99	147	0.99
9	146	0.97	150	0.97	155	0.98
10	110	0.96	105	0.97	106	0.97
11	131	0.98	108	0.98	106	0.98
12	126	0.99	123	0.99	119	0.99
13	119	0.99	100	0.98	98	0.98
14	147	0.96	143	0.98	120	0.97
15	122	0.98	112	0.99	98	1.00
16	131	0.99	126	0.99	121	0.99

The median value across the five hearts is presented

The HA transmural range in the mid-ventricle was found to be more robust to the effects of low resolution than at the apical or basal regions. Averaging across all regions, the range of the helix angle was 9° lower in the 3D–DS scheme, and 18° lower in the 2D–DS scheme than in the high-resolution data. In the basal regions (segments 1–6), the mean decrease was 14° in the 3D protocol, and 26° in the 2D protocol. In the mid-ventricular regions (segments 7–12), the mean decrease was 3° in the 3D–DS scheme, and 4° in the 2D–DS scheme. In the apical regions (segments 13–16), the mean decrease was 10° in the 3D–DS scheme, and 25° in the 2D–DS scheme.

**Fig. 2:**
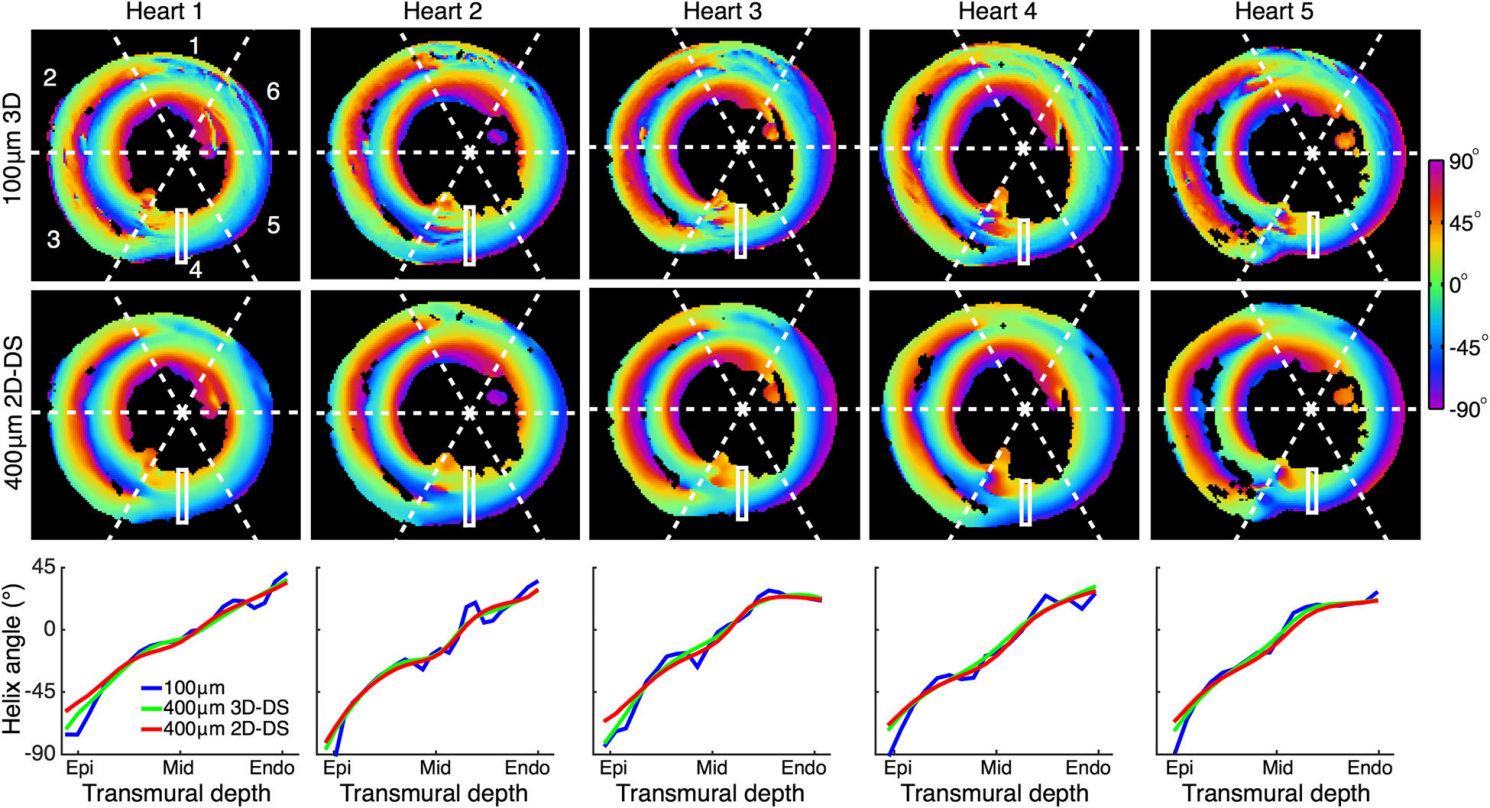
The effect of imaging resolution on transmural profiles in basal regions Top row: Helix angles in American Heart Association regions 1–6, with the representative profile of region 4 indicated, reconstructed at 100 μm. Middle row: the same slice at with 400 μm in-plane resolution, 1200 μm slice thickness (interpolated to 100 μm resolution prior to model fitting). Bottom row: The transmural profile in region 4 at the three resolutions

Figure 2 presents the effect of imaging resolution in the basal regions of the five hearts, with a focus on American Heart Association region 4. Region 4 was selected because it has a high HA rate-of-change (i.e. myocyte dispersion) at the sub-epicardium and a lower rate-of-change at the sub-endocardium. The lower resolution can be seen to cause a large over-estimation of the HA at the sub-epicardium, whereas the HA is largely unchanged by the loss of resolution at the sub-endocardium. In general, regions with greater dispersion exhibited greater bias at the lower resolution.

**Fig. 3:**
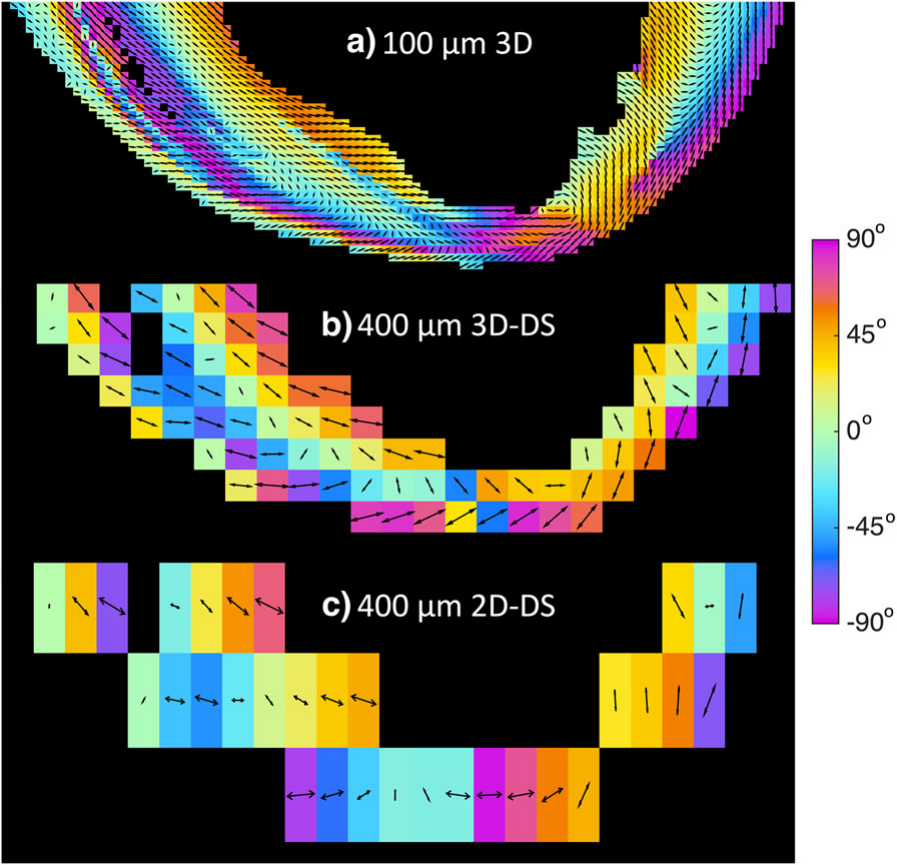
The effect of decreasing image resolution at the apex of heart #2. **a** An apical long-axis view of the 3D data acquired at isotropic 100 μm resolution. Voxels are coloured by helix angle. As helix angles are poorly defined at the apex, the projection of the primary eigenvector onto the image plane is also displayed. **b** The same slice as in a), re-sampled at isotropic 400 μm resolution. **c** The same data as in a-b), resampled to 400×400×1200 μm. The range of the helix angle and heterogeneity of vector orientations in the high-resolution image is progressively lost with decreasing resolution

An example of the effect of resolution on the HA at the apex is presented in Fig. 3. At 100 μm isotropic resolution, distinct populations of HA are visible. At 400 μm isotropic resolution, the range of HA was reduced and populations are merged. With a slice thickness of 1.2 mm, only the broad trends in HA transitions were preserved, while the range was further reduced.

### Experiment 2: Effects of ND and SNR

**Fig. 4:**
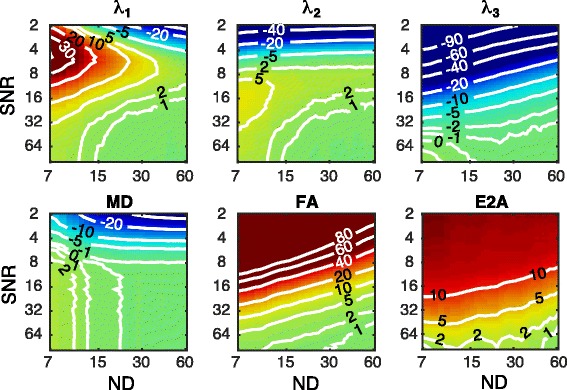
Accuracy of parameters (% error relative to ground truth) at varying ND and SNR_non-DW_. In general, λ_3_ requires higher SNR_non-DW_ and/or DW directions in order to achieve the same accuracy as λ_1_. λ_2_ is intermediate to λ_1_ and λ_3_ in terms of requirements. At SNR_non-DW_ < 5, mean ADC is underestimated as a result of the positive signal bias from the Rician noise, but has accuracy within ±1% for SNR_non-DW_ > 8 and ND > 12. FA is systematically overestimated, and has the most stringent requirements for accurate estimation. The E2A magnitude is also systematically overestimated. The contour lines represent the error compared to the ground-truth data in (%) for MD, FA and eigenvalues, and in (°) for angle maps

**Fig. 5:**
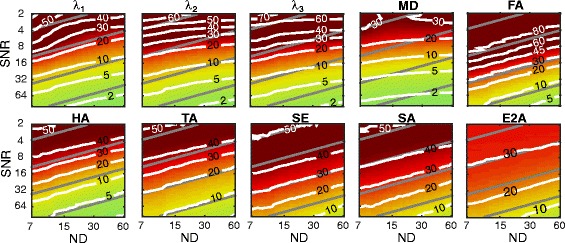
Precision of parameters with varying ND and SNR_non-DW_. The eigenvalues, MD, and FA are reported in % error relative to the ground truth, and the parametric angles are reported in degrees. Lines of equal scan time are shown in grey, and are spaced one order of magnitude apart. The precision of the ADC is better, and the precision of the FA is worse, than any of the eigenvalues individually. HA has better precision than TA, which is in turn better than the SE or SA. The E2A has better precision than the SE or SA, primarily as a result of evaluating only the magnitude. For a given scan time, the precision of the eigenvalues and ADC are optimised by maximising SNR_non-DW_ at the expense of ND. The precision of the FA and parametric angles are largely independent the SNR/ND trade-off. The contour lines represent the error compared to the ground-truth data in (%) for MD, FA and eigenvalues, and in (°) for angle maps

Figures 4 and 5 present the accuracy and precision of the principal eigenvalues, MD, and FA for the 2D–DS scheme, over a range of SNR_non-DW_ and ND values. Look-up tables for these figures are also provided [see Additional file 1]. The condition number of the direction sampling scheme [23], which gives an indication of system sensitivity, varied between 1.59 and 1.87 for the truncated data. Note that the condition number in the case of uniform sampling is 1.58 [24].

For SNR_non-DW_ > 6 and over a wide range of ND, our results indicate an over-estimation of λ_1_ and λ_2_, and under-estimation of λ_3_, leading to over-estimation of FA. No combination of SNR_non-DW_ or ND caused an underestimation of FA. In general, the accuracy of the FA was poorer than any of the eigenvalues individually. In contrast, the accuracy of the MD was better than the eigenvalues individually, and was within ±1% for SNR_non-DW_ > 8 and ND > 12. With fewer than 10 directions, the MD was systematically overestimated by more than 2% for all SNR_non-DW_ > 8. The accuracy of the HA, TA, SE, and SA (not shown) was within ±1° for all combinations of ND and SNR_non-DW_. The accuracy of the E2A magnitude had a positive bias, approaching zero for high SNR and ND.

The precision of the MD was better, and the FA was worse, than any of the eigenvalues individually. Unsurprisingly, the primary eigenvalue had the best precision, and the tertiary eigenvalue the worst. For a fixed scan time (displayed in Fig. 5 as parallel grey lines), the MD is optimised by maximising SNR at the expense of ND. This is also the case for the individual eigenvalues, albeit to a lesser extent. The precision of the FA is generally independent of the trade-off between ND and SNR, as the contour lines of the precision are parallel to the fixed scan time lines.

The precision of the HA was better than the one of the TA, and both were considerably better than the SE, SA, or E2A. The SA precision is marginally better than that of the SE. The E2A magnitude precision is better than the SE or SA, primarily as a result of the reduced range (i.e. 0–90°). Repeating the analysis on the signed E2A yields almost identical precision to the SE. The contour lines of all four parametric angles are approximately parallel to the fixed scan time lines, indicating that these parameters are largely independent of the trade-off between ND and SNR.

**Fig. 6:**
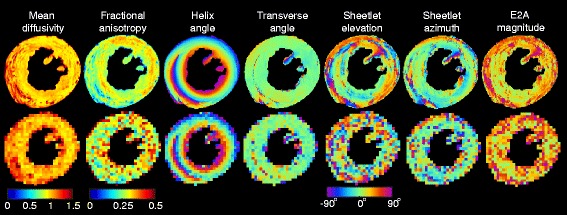
Comparison of parameter maps derived from the ground-truth data and a simulated in-vivo dataset. Top: Ground truth parameter maps reconstructed at 100 μm, ND = 61, no added noise. Bottom: Simulated in-vivo quality dataset, reconstructed at 400 μm, slice thickness = 1200 μm (2D–DS), SNR_non-DW_ = 30, ND = 10. Note that the mean diffusivity is in units of 10^−3^ mm^2^/s

Figure 6 presents a comparison of the ground truth parametric maps (top) with those derived from data with typical clinical parameters (bottom). The simulated clinical dataset was generated using the 2D multi-slice scheme, SNR_non-DW_ = 30, and ND = 10. The median (and interquartile range) of the simulated DTI parameters in the left ventricle are as follows: *λ*_1_ = 1.38 (0.01) × 10^−3^ mm^2^/s, *λ*_2_ = 0.99 (0.03) × 10^−3^ mm^2^/s, *λ*_3_ = 0.80 (0.02) × 10^−3^ mm^2^/s, MD = 1.06 (0.02) × 10^−3^ mm^2^/s, FA =0.27 (0.01). The expected bias (using Fig. 4) at this combination of SNR_non-DW_ and ND of *λ*_1_/*λ*_2_/*λ*_3_/MD/FA is +4.3/+4.5/−3.9/+2.1/+13.5% with respect to the ground truth data at the 2D–DS resolution, while the expected precision (using Fig. 5) is ±8.3/8.6/9.2/4.9/29.7%.

While HA and TA in the simulated images are robust to the lower SNR and ND, the SE and SA are more susceptible to the noise and limited number of directions. The precision of the HA, TA, SE and SA are ±14°, ±20°, ±31°, and ±28° respectively. The expected bias of the E2A magnitude is +8°, while the precision is ±24°.

## Discussion

There are many differences between clinical and ex-vivo cardiac DTI, including different pulse sequences, the use of fixative in ex-vivo imaging, and temperature differences, among others. However, the underlying compromise between SNR_non-DW_, ND, and resolution underpins all cardiac DTI. Ex-vivo imaging facilitates investigation of these relationships in ways that are not possible using only clinical data, and without the confounding factors of motion, strain and perfusion.

Based on averaged cardiomyocyte volumes in adult rat hearts [25] we estimate a cell density of 4.0 × 10^4^ cardiomyocytes per mm^3.^ Therefore, in the 100 μm ground truth data, each voxel contained approximately 40 myocytes, while in the simulated clinical data, each voxel contained approximately 7700 myocytes. The HA range was shown to be under-estimated at simulated clinical DTI resolution by an average of 18°, and by up to 30° in the basal and apical regions with respect to the high resolution CMR. This is in agreement with the recent work of Varray et al. [26], who demonstrated the relationship between resolution and helix angle range using synchrotron phase-contrast images with pixel size 3.5 μm to simulate various resolutions between 3.5 μm and 3.6 mm. The degree of under-estimation varies between regions of the heart, and is dependent on both the curvature of the tissue with respect to the imaging plane, and also the cardio-myocyte dispersion within each voxel. In mid-ventricular slices where the imaging plane is parallel to the surfaces of the heart, the mean decrease in helix angle range between 100 μm and 400 μm resolution was only 4°.

Of the parameters considered in this study, the MD was the least affected by ND. For a constant scan time, both the accuracy and precision of the MD is maximised by increasing SNR_non-DW_ at the expense of ND. This is not surprising, given that the MD does not require eigenvector accuracy, and can be computed with ND = 3. The precision of the individual eigenvalues was also maximised with maximum SNR_non-DW_, whereas the precision of the FA and parametric angles were largely independent of the trade-off between SNR_non-DW_ and ND. This is in agreement with simulations [27].

Mazumder et al. [28] compared cardiac DTI in-vivo acquired with various combinations of ND and NSA. They suggest that robust estimation of the HA is possible with combinations of ND/NSA of 12/6, 30/3, or 64/2. Of these, the shortest acquisition time was achieved with ND/NSA = 12/6 (i.e. maximising SNR at the expense of ND). A related study by Scott et al. [29] found that for ND ≥ 12, the effects of adding additional directions is small. A fundamental limitation of these two publications is that the experiments were performed in-vivo, which limited the achievable ground-truth image quality, and required motion and strain correction.

For all combinations of SNR and ND considered in this work, the biases of the eigenvalues maintain sorting order (i.e. bias in *λ*_1_ was the most positive, and the bias in *λ*_3_ was the most negative). This is due to the Rician noise affecting the eigenvalues unequally. As a result, the FA is invariably over-estimated. At typical clinical DTI settings, the bias in FA is +13.5%, which opposes the −7.1% bias arising from increased cardiomyocyte dispersion at the lower imaging resolution. This therefore suggests that FA should be interpreted in the context of resolution, SNR, and ND.

At typical clinical settings, we estimate the HA, TA, SE, and SA to have precisions of ±14°, ±20°, ±31°, and ±28° respectively. Our results indicate that the effect of noise on all parametric angles is a loss of precision only. The recent work of Scott et al. [10] found that the main effect of noise on HA was a loss of precision, whereas the effect of noise on the angle of the secondary eigenvector (E2A), which is related to the sheetlet elevation, is a loss of both precision and accuracy. Our results show that this apparent loss of accuracy arises from Scott et al. considering only the magnitude of the E2A, and thereby observing a noise-dependent bias.

In this work Rician noise was considered, as the images were acquired using a volume coil. When multiple coils are employed the noise profile takes a Chi-squared distribution [30]. However, given sufficient SNR (i.e. > 8), both can be approximated as having normally distributed noise. It is important to note that, for the number of directions typically employed in cardiac DTI (i.e. ≤12), the noise distribution in any single voxel will only be an approximation to the theoretical distribution. We repeated Experiment 2 using nonlinear least squares regression accounting for Rician noise (results not shown), and found predictably altered accuracy for SNR_non-DW_ < 8, but no improvement to the precision of any parameter for increasing SNR_non-DW_.

The differences in DTI-derived biomarkers between healthy and diseased can be subtle, particularly with respect to the inter-study variability. Comparing a study of 10 patients with hypertrophic cardiomyopathy [1] with a cohort of 10 healthy subjects imaged with a similar sequence [9], global MD was 6% lower (0.75 ± 0.15 vs. 0.80 ± 0.20) and global FA was slightly higher (0.61 ± 0.04 vs. 0.60 ± 0.04) in the hypertrophic hearts. Septal FA in the hypertrophic hearts was not found to be significantly different from that of the free wall. This is in contrast to the findings of Tseng et al. [31], who observed a 28% lower FA in the septum (0.56 vs. 0.78 in the free wall). In patients with heart failure (*N* = 3), MD was found to be 29% higher (1.8 ± 0.3 mm^2^/s, vs. 1.4 ± 0.2 × 10^−3^ mm^2^/s) than in healthy subjects (*N* = 20), whereas FA was 14% lower (0.24 ± 0.04 vs. 0.28 ± 0.06) [8].

Inter-study variation can be large, and can be attributed to the wide range of choices of imaging parameters. In normal hearts, FA values have been reported ranging from 0.29 to 0.43 [12, 13] in studies employing spin echo sequences, and from 0.40 to 0.61 [10, 16] in studies employing stimulated echo acquisition mode (STEAM) sequences. The differences arising from various diffusion sequences are compounded by those from the parameters discussed here, making direct FA comparisons across studies extremely difficult. One difference between spin echo and STEAM sequences is the diffusion time. Longer diffusion times in STEAM could in theory lead to better separation of the 2nd and 3rd eigenvalues, but at the cost of SNR efficiency from the 2-RR interval acquisition and the use of stimulated echoes [12]. We show here that low SNR leads to bias in DTI parameters, and the effects of partial volume obscuring finer structures in the down-sampled data. Independent validation notwithstanding, reliable sheetlet detection is feasible with both spin echo and STEAM approaches given sufficient SNR and image resolution. To improve clinical imaging and prospect for inter-study comparison, it is therefore essential to optimise and standardise DTI protocols.

While there are obvious differences between the rat and the human heart including size and heart rate, cardiomyocyte dimensions in the rat and human heart are similar, with lengths differing by <10% [32, 33], and it is reasonable to expect that the conclusions in this paper are equally valid for clinical DTI. Here we address the choice of ND, SNR and image resolution for a given scan time. Further important parameters would include the b-value [10], diffusion duration, and diffusion time. While precision can be improved by performing averaging over small regions of interest, the accuracy cannot.

We found marked and reproducible regional heterogeneity in parameters, such as SE and SA, reflecting discontinuous sheetlet arrangements, that was not necessarily evident in |E2A| values reported in ex-vivo pig hearts [34]. While different pulse sequences were used, we opine that the main factors contributing to the differences in heterogeneity are the higher image resolution in the current study and potentially large differences in sheetlet angles between mammals [17, 35]. Any heterogeneity due to fixation was minimised by comprehensive and extended exposure of hearts to fixative via perfusion and immersion.

This study was limited to the analysis of a single shell scheme with a b-value of 1000 s/mm^2^, which was close to the optimal value, defined by b = 1.1 / MD where MD ~1.1 × 10^−3^ mm^2^/s [36]. b-values in this range have been shown to minimise bias and absolute error in cardiac DTI parameters [10]. The DW sampling scheme was designed to be maximally uniform while permitting under-sampling, which is less desirable than the case where directions are chosen without this constraint (as is the case for real clinical data). As such, the condition numbers for our scheme were greater than the 1.58 of uniform sampling. However, we did not observe changes in accuracy or precision that may be attributed to fluctuations in the condition number of the scheme, for example between ND = 20, condition number = 1.74 and ND = 22, condition number = 1.59.

Even with higher order motion compensated DW gradients and/or double gating, clinical data can be corrupted by excessive or irregular motion that necessitates random rejection of corrupted images [9, 37]. Assuming a uniform sampling scheme, a complete loss of any one direction results in a condition number of 8.4 for ND = 8, 2.3 for ND = 10, 1.97 for ND = 12, and so on, approaching 1.58 for high ND. However, given that cardiac DTI is usually acquired with NSA ≥ 8, it is more likely that each direction will be acquired with a different number of viable images. Accounting for the variable NSA (and therefore SNR) in each direction using nonlinear fitting algorithms will likely improve tensor estimation compared to linear fitting approaches.

## Conclusions

The resolution, number of DW directions and number of averages in cardiac diffusion CMR experiments are important imaging parameters, which have non-trivial impacts on the diffusion tensor. Accurate and precise estimation of sheetlet angles is more demanding than estimating HA and TA, which in turn is more demanding than estimating MD. Our results indicate that with current clinical imaging protocols, the precision of sheetlet angles may be as poor as ±31°, and fractional anisotropy may be over-estimated by 13%. While this evaluation was performed in ex-vivo hearts, the findings are generalizable to the clinical setting that is subject to similar trade-offs.

## Additional file

Additional file 1: This spreadsheet contains look-up tables for Figs. 4 and 5. (XLSX 67 kb)

## Abbreviations

2D: Two-dimensional
3D: Three-dimensional
CMR: Cardiovascular magnetic resonance
DTI: Diffusion tensor imaging
DW: Diffusion-weighted
E2A: Secondary eigenvector angle
FA: Fractional anisotropy
Gd: Gadolinium
HA: Helix angle
MD: Mean diffusivity
ND: Number of directions
NSA: Number of signal averages
SA: Sheetlet azimuth
SE: Sheetlet elevation
SNR: Signal-to-noise ratio
STEAM: Stimulated echo acquisition mode
TA: Transverse angle
TR: Repetition time
